# Editorial: Translation and Processing of Light by the Non-image Forming Visual System—Context, Mechanisms and Applications

**DOI:** 10.3389/fneur.2021.727849

**Published:** 2021-08-27

**Authors:** Shadab A. Rahman, Fabian-Xosé Fernandez, Manuel Spitschan

**Affiliations:** ^1^Division of Sleep and Circadian Disorders, Departments of Medicine and Neurology, Brigham and Women's Hospital, Boston, MA, United States; ^2^Division of Sleep Medicine, Harvard Medical School, Boston, MA, United States; ^3^Department of Psychology, University of Arizona, Tucson, AZ, United States; ^4^BIO5 and McKnight Brain Research Institutes, University of Arizona, Tucson, AZ, United States; ^5^Department of Experimental Psychology, University of Oxford, Oxford, United Kingdom; ^6^Nuffield Department of Clinical Neurosciences, Sleep and Circadian Neuroscience Institute (SCNi), University of Oxford, Oxford, United Kingdom; ^7^Centre for Chronobiology, Psychiatric Hospital of the University of Basel, Basel, Switzerland; ^8^Transfaculty Research Platform Molecular and Cognitive Neurosciences, University of Basel, Basel, Switzerland

**Keywords:** light, non-visual effects of light, non-imaging-forming pathways, ipRGCs, melanopsin, circadian rhythms, light exposure, intermittent light exposure

## It's About Light

We have the great pleasure of editing a special issue concerning the sensory integration of visible light across different exposure durations by the non-image forming photobiological system including the central circadian system. Throughout the past several decades, the circadian field has seen extraordinary progress. Sixty years have passed since the seminal Cold Spring Harbor Symposium on Biological Clocks. Forty have passed since the finding that light gates melatonin secretion in humans, while only 20 years separates us from the discovery of intrinsically photosensitive retinal ganglion cells (ipRGCs) and their expression of the short-wavelength photopigment, melanopsin. The field is once again at an exciting watershed, this time created by advances in semiconductor light emission technologies that offer the ability to “tune” multiple characteristics of photic stimulation (e.g., intensity, duration, spectrum, exposure pattern), thereby enabling us to engineer human-centric lighting that is designed for not just optimal vision but also mental and physical health. In observance of this watershed (which is still unfolding), we have drawn together an issue of 16 articles covering a wide range of model organisms and considerations for what constitutes “biologically active” light, how to quantify, measure, and report it, as well as applications of biologically-active exposure patterns that might improve health outcomes. The issue is thus comprised around the following topics.

## Approaches to Defining Light Exposure

Kicking off the volume with a didactic tutorial, Spitschan introduces the concept of time-varying light (from milliseconds to hours), its properties, and how to describe its ambient/environmental and exposure characteristics during laboratory investigations assessing the impacts of light on sleep and circadian function. Important issues are raised in the specification of these domains, including: (1) the challenges with calibrating the temporal output of light generators operating on the sub-second timescale, and (2) the possibility of non-linear distortions occurring in light emission across the exposure duration based on input variability (e.g., the output spectra of LEDs can shift by a few nanometers depending on the electrical driver, creating potential artifacts in photoreceptor stimulation). The mini-review ends with a call for more consistency in scientific communication to assure that current reports are “future-proofed” as changes in lighting technology increasingly fuel investigations of non-image forming responses to new and enhanced human centric lighting devices.

Understanding the biological potency of light exposure requires accurate metrology. Schlangen and Price introduce fundamental concepts related to the measurement and characterization of emission sources and of the lighting environment in general, with reference to the recent International Standard CIE S 026/E:2018. This standard recommends grounding predictions about the wake or sleep-promoting effects of light exposure on its activation of each of the five photoreceptor types. The authors close their article by contributing new data illustrating how the melanopic content of light can be derived from everyday light sources, including daylight, and how such assessments, if standardized, can be used to better understand and improve the impact of lighting on human well-being.

Three author teams examine the *bench-to-bedside* extension of laboratory findings on the biological effects of light exposure into everyday application. First, Stefani and Cajochen review current advisories on ambient lighting that are intercalated across various societal frameworks, from academic recommendations to formal “best-practices” guidelines and workplace-industry ordinances. They conclude that in many cases existing standards are based on visual lighting needs for a given occupational setting (e.g., minimum levels of illuminance required for performance or color rendering quality needed to ensure visual comfort) and have yet to fully integrate an understanding of the influence of light exposure on non-visual physiological responses (e.g., minimum levels of melanopic-EDI required for alertness across the workday). Rounding out their perspective, the authors discuss several laboratory experiments suggesting that dynamic lighting mimicking changes in natural daylight can improve circadian and sleep health while maintaining adequate illumination for work-related visual tasks. As such, regulations looking to integrate visual and non-visual lighting considerations can be credibly developed, as embodied in several design guidelines for the built environment already emerging in North America and Europe.

Next, targeting practitioners, Houser and Esposito outline a five-step process for imbuing current scientific knowledge on the non-visual effects of light exposure into (physiologically relevant) human-centric lighting design. These steps include characterizing the lighting application, determining the habitual or desired sleep-wake schedule of the occupants and their sleep needs, and staying informed of building specifications that optimize functional balance between the visual, arousal, and circadian systems. Like Stefani and Cajochen, the authors conclude that architecture lighting (with the proper control systems) can be engineered to deliver biologically potent light during the day and low-potency light at night, all while balancing traditional factors such as color quality, flicker, and visibility.

Soler and Voss discuss principles for the application of emergent lighting technology including LED lighting from an industry perspective. They summarize the influence of important variables such as spectral power distribution and spatial geometry of the built environment that require careful consideration when designing indoor lighting solutions. Incorporating such principles at the design stage has the potential to maximize the ability to create “biologically” brighter days and darker nights. Their piece complements the reviews from Stefani and Cajochen, and from Houser and Esposito providing insights relevant for industry practitioners.

Taking a broad comparative-translational perspective, from rodents to humans, Walbeek et al. review evidence that naturalistic intensities of light at night (nLAN) provide important information that orients mammalian circadian and neuroendocrine physiology. The authors note that the terminology used in the field to date has been wildly inconsistent, with the term “dim light” being used to describe light levels ranging seven orders of magnitude (0.0001 to 500 lux). When properly defined and calibrated to intensities approximating the moon and stars, extant data suggest that nLAN can influence common parameters measured within circadian oscillations (e.g., free-running period and waveform), function as a zeitgeber, and produce direct non-visual effects on melatonin secretion and pupil constriction. While the properties of nLAN have been documented exclusively in rodents, there is a reasonable expectation that they will have translational potential for humans—imparting either analogous effects to those in animal models or more subtle effects on the plasticity of light responses. The authors close their discussion with a consideration of some of the challenges involved with measuring nLAN and the need to possibly rethink the current human-centric lighting recommendation that nights should be *completely* dark.

## Model Systems, Mechanistic Insights, and Benchmarking the Effects of Light Exposure

Understanding the effects of light exposure on the circadian pacemaker requires understanding the molecular and cellular mechanisms underlying the encoding of stimulus strength and the circuitry that integrates the signal from the retina to the pacemaker. In this issue, four contributions provide unique insights into these mechanisms and pathways, each using a different model system. First, Tabuchi et al. review the signaling biology that connects oscillations of the molecular clock with daily changes in membrane excitability in *Drosophila* timekeeping neurons. At the cross-roads of each is cryptochrome (CRY), a photosensitive molecule in flies that is embedded within the circadian transcriptional–translational machinery and an effector of the potassium ion channel β-subunit redox sensor. In addition to CRY, several other molecular clock constituents have been shown to modulate the neural activity of fly timekeeping neurons via interactions with ligand-gated and voltage-dependent/independent channels, channel-binding proteins, and subunits of the electrogenic ion pump. The authors suggest that the generalization of this crosstalk motif—between clock gene oscillations and daily changes in neuronal excitability—offers a new paradigm by which to study the effects of light exposure on sleep-wake rhythms, cognition, and neurological disorder.

Hannibal provides an intriguing and in-depth review of the comparative neuroanatomy of the retinohypothalamic tract and its role in mediating non-image forming (NIF) responses to light exposure in mammals. Drawing insights from studies on various genetic models and unique neuroanatomic features (e.g., 90% of retinal ganglion cells in the mole rat express melanopsin compared to 1% of the cell population in most laboratory rodents), Hannibal elucidates the molecular and neuronal pathways underlying NIF responses to light exposure. A spotlight is placed on neurotransmitter systems connecting the front-end of circadian photoreception to the hypothalamus, including those using the neuropeptide PACAP. A perspective is also provided on the harmful effects of light exposure at night on humans and other animals.

Next, Wong and Fernandez zoom out of the picture to discuss how intermittent light exposure through a sequence of millisecond light flashes is encoded as a coherent stimulus by the circadian system. They place the signaling of time at twilight within a theoretical framework that suggests the pivotal role of cones in this process, tying together different ideas from Gestalt theory and bringing them into a non-image forming context, thus generalizing principles of efficient sensory coding from one to the other. To support their “Cone Sentinel” model, the authors provide the first experimental data showing that ipRGC responses to short light flashes are dependent on extrinsic synaptic relays from classic photoreceptor cells and not intrinsic melanopsin expression. These data offer the unique perspective that flash protocols optimizing the stimulus-response characteristics of cone photoreception have the potential to be used clinically to treat sleep/circadian disorders and other medical conditions with an underlying circadian impairment.

While light exposure can be modulated to examine how the circadian system responds to it, it can also be used to disrupt the system—or eliminate it entirely. To do so, Ruby introduces the disruptive phase shift (DPS) protocol, a simple manipulation of ambient lighting that renders animals (Siberian hamsters) arrhythmic without altering their genome, or the tissue and developmental integrity of the brain's clock, the suprachiasmatic nucleus (SCN). The DPS model has the advantage of studying arrhythmia in a “natural” state within the brain. In this model, the SCN circuitry remains normally intact (albeit dysfunctional) and thus can influence other structures to which it projects and receive input from networks returning from those structures. In that regard, Ruby offers an inroad to studying the neurophysiology of circadian dysrhythmia in an animal the way it is most likely to present in a human. DPS hamsters show severe impairments in declarative memory that are associated with fragmentation of electroencephalographic theta oscillations, as well as altered GABAergic and cholinergic signaling in the hippocampus. These phenotypes may point the way toward developing pharmacological treatments for circadian disturbances related to jetlag and those incurred with chronic disease.

Finally, Mure provides a state-of-the-art review on what is currently known about the functional properties of human ipRGCs, an extremely difficult system in which to conduct electrophysiological recordings. The article takes an integrative stance, reviewing transcriptomic and morphological phenotypes that may distinguish various subtypes (GM1, M1-M4) and brain connectivity maps generated via PACAP (pituitary adenylate-cyclase-activating polypeptide) immunohistochemistry. By and large, the light-response characteristics of human ipRGCs are similar to their variants in rodents and primates. In each case, the spectral sensitivity of the cells peaks around ~460 nm, with kinetic properties of intrinsic responses exhibiting a slow, sustained period of activity during stimulation that continues after light exposure. The author concludes the article by reviewing how altered ipRGC response kinetics or cell loss might provide insight into pathological mechanisms underlying aging-related neurodegenerative diseases such as Alzheimer's and Parkinson's disease.

## Precision Lighting: Applications of Tunable Light Exposure

Current lighting technology allows tuning of various stimulus characteristics, including timing, intensity, spectrum, and pattern. Kawasaki et al. investigate the extent to which morning bright light (~10,000 lux, 30 min per day for 4 weeks) influences metrics related to sleep and non-visual physiological function in patients with glaucoma, an optic neuropathy that results in ipRGC loss. Post-intervention, those exposed to light therapy reported better subjective sleep quality on a Likert scale. The treatment group also demonstrated increased melanopsin-dependent, post-illumination pupil responses that correlated with common measures employed to quantify the robustness of daily ~24-h oscillations in physical activity assessed using actigraphy. The authors concluded that scheduled bright light exposure may be a cost-effective strategy to improve sleep, circadian entrainment, and general sense of well-being in patients with glaucoma.

Using an overnight (2-day) in-laboratory randomized trial in healthy college-aged young adults, Grant et al. examined whether daytime exposure to LED task lighting with different spectra conferring different melanopic illuminance (melEDI) can affect neurobehavioral performance. Their key finding was that long duration (8 h) light exposure with higher melanopic content was associated with significant improvements in working memory and processing speed (as assessed using a 2-min addition task) and improved procedural learning in a motor sequence task. These data provide preliminary evidence indicating that the incorporation of lighting with higher melEDI in the built environment (i.e., a modification resulting in few changes in perceived lighting) may improve neurobehavioral performance in the classroom in college-aged young adults with a naturally restricted sleep schedule.

An additional two primary reports from randomized trials elucidate how spectrally tuned light exposure affects the brain and behaviors associated with underlying mood, cognition, and arousal. First, Raikes et al. examined the extent to which daily morning short-wavelength (blue) light therapy over 6 weeks, compared to therapy with longer wavelength (amber) light exposure, changes regional functional connectivity in the brain and ratings of subjective sleepiness in patients recovering from a mild traumatic brain injury (mTBI). At the conclusion of the 6-week treatment, improvements were observed in gray matter volume in the right thalamus and functional connectivity between regions of interest related to attention, somatomotor, and visual networks as well as the default mode network (e.g., those interconnecting the thalamus with the prefrontal and orbitofrontal cortices) in patients receiving blue light exposure as compared to amber light exposure. Furthermore, these morphological and functional connectivity improvements were associated with less daytime sleepiness reported with the Epworth Sleepiness Scale. The authors concluded that blue light therapy might be a non-invasive way of improving sleep and thereby improving functional outcomes after mTBI.

Using a shorter duration (30 min) single-exposure administration of blue and amber light, Alkozei et al. examined changes in indices of functional and directed connectivity between the amygdala and the left dorsolateral prefrontal cortex, and the association of these outcomes with affect. The authors report that individuals receiving blue light exposure had greater bidirectional information flow between these two regions about an hour after the light exposure had stopped. This enhanced connectivity was associated with decreased negative affect. These results suggest that short-interval blue-light therapy can facilitate the engagement of cognitive control strategies needed to regulate arousal/mood and provide some mechanistic insight into why conventional bright light therapy during the winter months might improve depressive symptoms in people with seasonal affective disorder.

Finally, Figueiro and Leggett review the evidence for the beneficial effects of intermittent light exposure in Alzheimer's disease (AD). Two strategies are discussed relating to (1) the delivery of 40-Hz flickering light to promote gamma oscillations and gamma-related clearance of the amyloid beta (Aβ) peptides contributing to plaque formation in the AD brain and (2) the delivery of timed millisecond flashes during sleep to induce phase-resetting responses that may help reinforce entrainment of sleep-wake schedules, thereby also facilitating Aβ clearance (e.g., the recently discovered glymphatic system has been shown to remove Aβ specifically during sleep). Overall, the authors point out that these two strategies are not mutually exclusive when considering how intermittent light stimulation can be parlayed for the treatment of AD-related phenotypes and envision the development of personalized light therapy devices that might one day achieve both.

This volume touches on only a sliver of the developments that are currently permeating the field of circadian photobiology. Nevertheless, we hope that we have been able to capture some of the current “frontier” thinking in editing this issue, thus inching a little beyond the *zeitgeist*.

## Author Contributions

All authors listed have made a substantial, direct and intellectual contribution to the work, and approved it for publication.

## Conflict of Interest

SAR holds patents for Prevention of Circadian Rhythm Disruption by Using Optical Filters and Improving sleep performance in subject exposed to light at night; SAR owns equity in Melcort Inc.; has provided paid consulting services to Sultan & Knight Limited, Bambu Vault LLC, Lucidity Lighting Inc.; and has received honoraria as an invited speaker and travel funds from Starry Skies Lake Superior, University of Minnesota Medical School, PennWell Corp., and Seoul Semiconductor Co. Ltd. These interests were reviewed and managed by Brigham and Women's Hospital and Partners HealthCare in accordance with their conflict of interest policies. The remaining authors declare that the research was conducted in the absence of any commercial or financial relationships that could be construed as a potential conflict of interest.

## Publisher's Note

All claims expressed in this article are solely those of the authors and do not necessarily represent those of their affiliated organizations, or those of the publisher, the editors and the reviewers. Any product that may be evaluated in this article, or claim that may be made by its manufacturer, is not guaranteed or endorsed by the publisher.

